# Translation and psychometric validation of the Intent-to-Aid survey for bystander response among French-speaking CPR-trained populations

**DOI:** 10.1016/j.resplu.2026.101400

**Published:** 2026-06-29

**Authors:** Marco Pedrotti, Baptiste Lucien, Philippe Terrier, Jérémy Torrent, Sandrine Dénéréaz, Thierry Spichiger, Christophe Bélet, Jeffrey L. Pellegrino

**Affiliations:** a Haute École Arc Santé, HES-SO University of Applied Sciences and Arts Western Switzerland, Espace de l’Europe 11, 2000 Neuchâtel, Switzerland; b ES ASUR, Vocational Training College for Registered Paramedics and Emergency Care, En Budron C8, 1052 Le Mont-sur-Lausanne, Switzerland; c Fondation RéaJura Coeur, Voyeboeuf 13, 2900 Porrentruy, Switzerland; d Emergency Management & Homeland Security, College of Health & Human Sciences, University of Akron, Polsky Bldg 315, Akron, OH 44325-4304, United States of America

**Keywords:** Out-of-hospital cardiac arrest, Bystander cardiopulmonary resuscitation, Resuscitation education, Behavioral determinants, Psychometric validation

## Abstract

**Background:**

Bystander cardiopulmonary resuscitation (CPR) and automated external defibrillator (AED) use are critical determinants of survival after out-of-hospital cardiac arrest (OHCA). These behaviors are influenced not only by knowledge and skills but also by affective determinants such as confidence, attitudes, and social norms. The Intent-to-Aid (I2A) survey was developed to measure these constructs. The European Resuscitation Council (ERC) has highlighted the need for tools to evaluate resuscitation education outcomes in non-English-speaking populations to support equitable access to training.

**Objective:**

To translate, culturally adapt, and psychometrically validate the I2A for French-speaking CPR-trained individuals.

**Methods:**

The I2A was translated using forward–backward procedures, expert review, and cognitive debriefing. In a cross-sectional study of French-speaking CPR-trained adults in Switzerland (*N* = 378), confirmatory factor analyses (CFA) were conducted at parcel and item levels. Internal consistency was assessed using Cronbach’s alpha and McDonald’s omega, and associations between Theory of Planned Behavior constructs were examined to assess theoretical coherence.

**Results:**

The original four-factor structure (Attitudes, Norms, Confidence, Intentions) was replicated in parcel-based CFA, demonstrating excellent overall model fit. Item-level CFA supported the same structure after removing one item with extreme ceiling effects. Internal consistency ranged from acceptable to excellent (*α* = 0.66–0.87; *ω* = 0.75–0.90). Confidence and Norms explained most variance in Intentions, highlighting potential targets for resuscitation education.

**Conclusions:**

The I2A-French demonstrates robust structural validity, satisfactory reliability, and theoretical coherence in highly trained CPR populations, supporting its use for evaluating affective determinants of bystander CPR response.

## Highlights

• First French validation of the Intent-to-Aid survey for bystander CPR.
• Replicates the four-factor structure of attitudes, norms, confidence, intentions.
• Confidence and social norms strongly predict intention to provide CPR.
• Tool enables evaluation of affective outcomes in resuscitation education.

## Introduction

Survival after out-of-hospital cardiac arrest (OHCA) strongly depends on rapid recognition, early cardiopulmonary resuscitation (CPR), and early defibrillation by lay responders. Although efforts to expand community CPR training have increased, bystander intervention rates remain highly variable across regions and populations.^1^, ^2^ However, most evaluations of CPR and automated external defibrillator (AED) training still focus primarily on cognitive and psychomotor outcomes. Fewer studies address affective determinants of helping behavior or persistent inequities in access to resuscitation training across socioeconomic, racial, and demographic groups, as highlighted by recent ILCOR (International Liaison Committee on Resuscitation) analyses.^2^ Most educational studies continue to assess proximal outcomes such as knowledge acquisition, skills performance, and CPR quality metrics, while fewer studies evaluate behavioral intention or real-world helping behaviors.^3^, ^4^, ^5^ Yet, as Miller and Pellegrino^6^ demonstrated in developing the Intent to Aid (I2A) survey, the affective domain plays a central role in shaping lay responders’ intention to provide first aid in emergencies. Grounded in Theory of Planned Behavior, the I2A captures four key constructs (Attitudes, Norms, Confidence, Intentions) and has shown promising psychometric properties in English-speaking lay responder populations.^6^ The original I2A thus offers health education researchers and curriculum developers a theory-based instrument to assess the affective impact of first aid and CPR/AED training and to differentiate the effects of various pedagogical modalities on learner intent to help.^6^

To date, however, the I2A has not been translated or validated for use in other languages, nor has its structure been examined in trained responder populations such as first responder volunteers and health-professions students. In Switzerland, first responder volunteers are lay individuals trained in CPR and AED who are alerted by emergency medical dispatch (144) via a smartphone application to nearby suspected out-of-hospital cardiac arrests. Switzerland provides a relevant setting for this adaptation due to widespread CPR training initiatives, including mandatory BLS training for driver licensing and expanding first responder networks. In this context, first responder volunteers initiate BLS within three minutes of collapse in approximately 52% of cases.^7^ At the same time, the high training density and relative cultural homogeneity of Swiss CPR-trained populations may limit the generalizability of psychometric findings to less-trained or more diverse French-speaking settings. First responder volunteers and nursing students represent particularly relevant populations for this evaluation, as they frequently encounter simulated or real emergencies and often act as CPR trainers or community advocates.

The present study extends the work of Miller and Pellegrino^6^ by translating and adapting the Intent-to-Aid (I2A) survey for French-speaking CPR-trained populations and evaluating its measurement properties. By providing a validated instrument to assess affective determinants of bystander response, this study aims to support the evaluation and design of resuscitation education programs.

## Methods

### Design

This study was conducted as a cross-sectional psychometric validation study aimed at translating, culturally adapting, and evaluating the measurement properties of the Intent-to-Aid (I2A) survey in French-speaking CPR-trained adults. Structural validity was defined a priori as the primary outcome, followed by internal consistency analyses and examination of theoretical coherence within a Theory of Planned Behavior framework. Confirmatory factor analysis (CFA), a statistical approach used to examine whether questionnaire items reproduce an expected theoretical model, was conducted to evaluate structural validity.

The study design and reporting were informed by COSMIN recommendations.^8^ In addition, reporting of the cross-sectional design adhered to the STROBE checklist for observational studies.^9^ The translation and cultural adaptation process followed established cross-cultural adaptation recommendations to ensure semantic, conceptual, and contextual equivalence.^10^

### Ethics

The study protocol was submitted to the Cantonal Research Ethics Committee of Vaud (CER-VD; Req-2025-00851). The Committee confirmed that the project does not fall within the scope of the Swiss Federal Act on Research involving Human Beings (HRA) and does not require formal ethical approval. All procedures complied with applicable Swiss data protection regulations in alignment with the Declaration of Helsinki. Participation was voluntary, anonymous, and based on electronic informed consent.

### Translation and cultural adaptation

Two independent forward translations into French were performed by professional translators, followed by synthesis into a single version. Two independent back-translations into English were then conducted by native English speakers blinded to the original instrument. An expert committee, including the original instrument developer, reviewed all versions to ensure semantic, conceptual, and cultural equivalence.

The pre-final French version was pilot-tested using cognitive debriefing in two subsequent samples of French-speaking participants (*N* = 22 and *N* = 26, respectively), separate from each other and from the final validation sample. Participants included nurses, paramedics, and nursing students. During cognitive debriefing, participants were asked to identify items they considered unclear or difficult to understand. After the first round, 14 items received more than 20% of “unclear” responses and were revised by the expert committee to improve clarity and cultural appropriateness. A second round of cognitive debriefing was then conducted using the revised version. Following this second round, only one item still exceeded the predefined 20% threshold for “unclear” responses and was subsequently modified slightly, resulting in the final I2A-French version (see supplementary material).

### Participants and recruitment

Participants were recruited in Switzerland from two groups proficient in French: (1) first responder volunteers, and (2) nursing students in their 1st–3rd year. Inclusion criteria were age ≥18 years and prior CPR training. Participants were recruited via institutional email, in-class announcements, and organizational communication channels. No financial compensation was provided. Data were collected via REDCap on secure institutional servers.^11^, ^12^ Participation was voluntary, anonymous, and preceded by electronic informed consent. The survey did not ask participants to identify any group membership. Given the voluntary nature of participation, self-selection bias toward highly motivated individuals cannot be excluded.

### Measures

The I2A-French assesses four Theory of Planned Behavior constructs: Intentions, Attitudes, Social Norms, and Confidence (self-efficacy).^6^ Thirty-four items are rated on 5-point Likert-type scales (1–5). Subscale scores were computed by summing item responses, with higher scores indicating higher levels of the construct.

### Sample size

Sample size was determined a priori based on recommendations for CFA.^13^, ^14^ A minimum sample of 300 participants was targeted to ensure stable estimation of factor loadings and model fit indices in CFA analyses. The final sample size (*N* = 378) ensured an adequate participant-to-parameter ratio for stable CFA estimation at both parcel and item levels.

### Data analysis

All CFA models were estimated using methods appropriate for missing data and ordinal Likert-type responses. For subscale scoring and descriptive statistics, missing values (≤1.4% per item) were imputed using item means, consistent with the original validation study.^6^ Cronbach’s alpha and McDonald’s omega were used as complementary indicators of internal consistency reliability. Cronbach’s alpha was computed on imputed subscale item responses, given the very low proportion of missing data. Because item loadings were not fully homogeneous across subscales, McDonald’s omega was also estimated to provide a complementary reliability indicator. In line with the multidimensional structure of the I2A, internal consistency was evaluated separately for each subscale rather than for a total score.

A CFA was conducted using the parcel structure proposed by Miller and Pellegrino^6^ to evaluate whether the original latent model could be replicated in the French version of the I2A survey. Parceling was retained to allow direct replication of the original validation study. Parcels were computed as the mean score of their respective items, following the procedure described by Miller and Pellegrino.^6^ To allow direct comparison with Miller and Pellegrino,^6^ we also computed the Goodness-of-Fit Index (GFI). Although GFI is no longer recommended in contemporary guidelines due to its sensitivity to sample size and model complexity,^15^, ^16^ we report it here for completeness.

A second item-level confirmatory factor analysis was conducted using methods appropriate for ordinal Likert-type questionnaire data. The initial model included all 34 questionnaire items, and standard model fit indicators were evaluated according to established recommendations.^13^, ^17^ The R code and raw data are provided as supplementary materials.

## Results

Recruitment took place in November 2025, and 380 survey responses were obtained between November 15th and 27th. Of the 380 submissions, two cases were excluded (one empty questionnaire and one participant reporting no prior CPR training), resulting in a final analytic sample of 378 participants. The total number of individuals invited could not be precisely determined due to open distribution channels; therefore, a response rate could not be calculated. Median completion time was 6 min and 17 s. Half of the sample reported ≥5 previous CPR courses. Table 1 displays participant characteristics.

**Table 1:** Participant characteristics.

	***n***	***%***
**Previous CPR courses taken**		
1	33	8.7
2	58	15.3
3	56	14.8
4	35	9.3
≥5	190	50.3
No response	6	1.6
**Ethnic background (multiple responses allowed)**		
African	4	1
American Indian	3	0.8
Asian	3	0.8
Caucasian	362	94
Other	10	2.6
No response	3	0.8
**Age (years)**		
18–20	23	6.1
21–30	83	22
31–40	70	18.5
41–50	75	19.8
51–60	78	20.6
>60	39	10.3
No response	10	2.6
**Education**		
No completed education	10	2.6
Upper secondary education (vocational certificate, general education school, or equivalent)	126	33.3
Higher professional education (professional college, federal diploma or equivalent)	88	23.3
Bachelor’s degree (University/University of Applied Sciences/University of Teacher Education)	71	18.8
Master’s degree (University/University of Applied Sciences/University of Teacher Education)	64	16.9
Doctoral degree (PhD)	10	2.6
Prefer not to answer	9	2.4
**Sex**		
Female	172	45.6
Male	206	54.4
Other	0	0
No response	0	0

*Note:* CPR = cardiopulmonary resuscitation. *N* = 378. Ethnic background was assessed using a multiple-response question; therefore, percentages were calculated using the total number of ethnic background responses (*n* = 385) as the denominator rather than the total number of participants (*N* = 378).

### Replication of the original four-factor structure (parcel-based CFA)

**Fig. 1:**
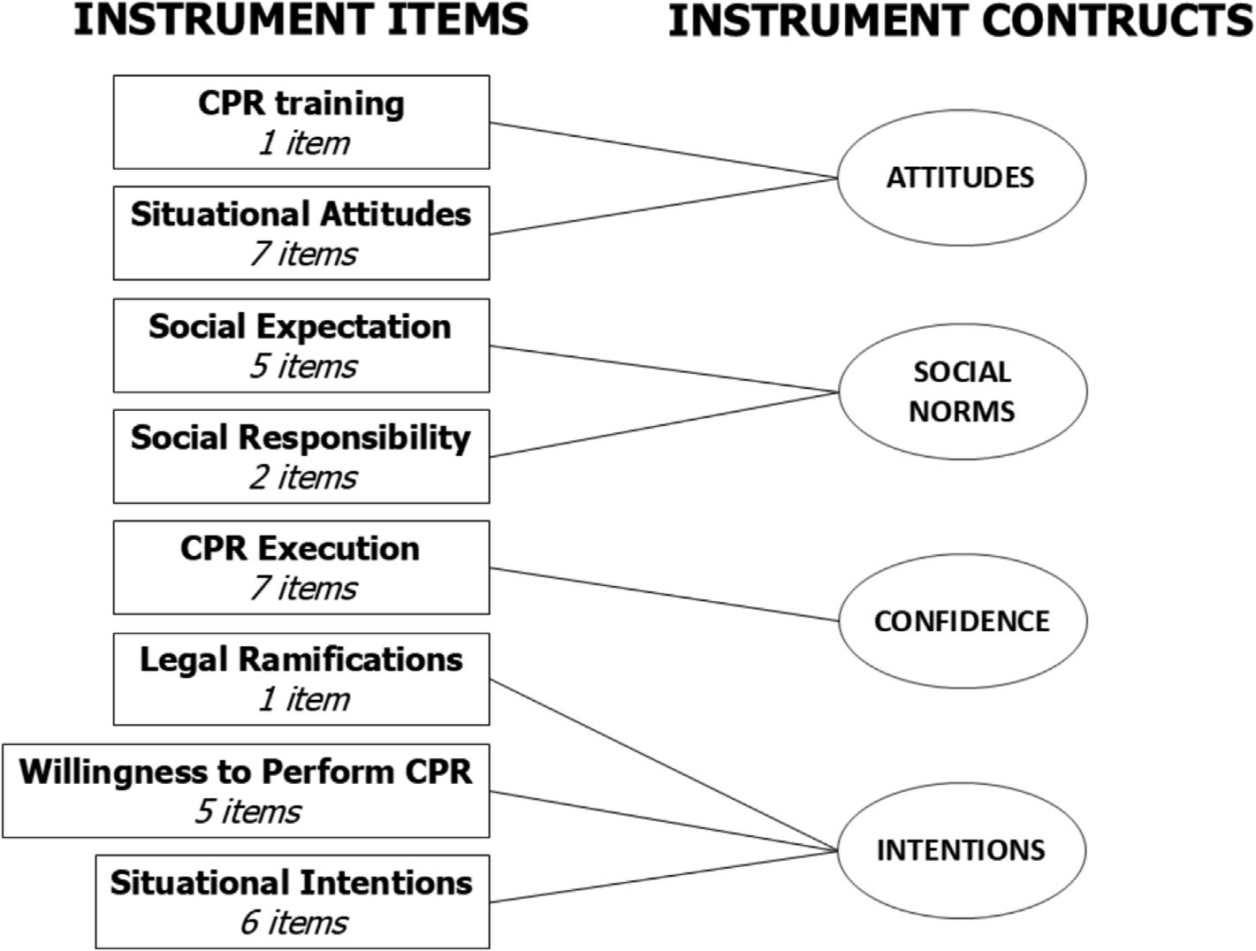
**Confirmatory factor analysis (CFA) item domain loading by Theory of Planned Behavior construct. Adapted from Miller and Pellegrino with permission.**^6^**Squared items represent measure questions domains (parcels), elliptical items represent latent Theory of Planned Behavior constructs. Solid lines indicate factor loading. No cross-loadings were allowed**.

As a first step, we tried to replicate the structure proposed by Miller and Pellegrino.^6^ The four-factor structure – Attitudes, Social Norms, Confidence, and Intentions – showed excellent model fit (Table 2). Standardized factor loadings were moderate to high for most parcels (*λ* = 0.52–0.86), whereas the CPR training domain (single-item parcel; see Fig. 1) showed a lower loading (*λ* = 0.32). All latent factor covariances were positive and statistically significant (*p* < 0.01), consistent with the theoretical relationships underlying the original English I2A framework. This analysis confirms that the structure reported by Miller and Pellegrino^6^ is successfully reproduced in the French translation (Fig. 1). Based on this result, a more detailed item-level CFA was conducted.

**Table 2:** Confirmatory factor analysis.

**Parcel-based CFA – Replication of Miller and Pellegrino**^6^			
***Fit parameter***	***I2A-English***^6^	***I2A-French***	***Fit criteria***
*χ*^2^(df), *p* value	15.73(13), *p* = 0.264	23.66(15), *p* = 0.071	*p* > 0.05
RMSEA	0.033	0.039	<0.05
SRMR	0.049	0.024	<0.05
CFI	N/A	0.992	≥0.90
TLI	N/A	0.986	≥0.90
GFI	0.981	0.980	≥0.90

**Item-level CFA (WLSMV)**			

***Fit parameter***	***I2A-Frenchinitial model***	***I2A-Frenchmodified model***	***Fit criteria***

*χ*^2^(df), *p* value	1721(521), *p* = 0.000	1653(489), *p* = 0.000	Not interpreted
RMSEA	0.083	0.084	<0.10
SRMR	0.090	0.091	<0.10
CFI	0.982	0.975	≥0.90
TLI	0.981	0.973	≥0.90

*Note*. Model fit indices evaluate how well the observed questionnaire responses correspond to the expected theoretical structure, with higher CFI/TLI and lower RMSEA/SRMR values indicating better fit. RMSEA = root mean error of approximation; SRMR = standardized root mean square residuals; CFI = comparative fit index; TLI = Tucker-Lewis index; GFI = goodness-of-fit index; N/A = not available.

### Item-level CFA

The overall fit of the model was acceptable but not optimal (Table 2, initial model). All factor loadings were statistically significant (*p* < 0.001). However, item Q1_3 (*I am willing to help another person I know in a first aid emergency*) presented severe psychometric issues: strong ceiling effect (94.7% of responses at the maximum value), very low variance leading to a Heywood case (negative residual variance), inflated standardized factor loading and unstable factor variance for Intentions. According to methodological guidelines,^13^ items with near-zero variance and inadmissible residual variances should be removed or revised. The decision to remove Q1_3 was based on predefined psychometric criteria (extreme ceiling effect, inadmissible residual variance, and model instability) rather than post-hoc modification indices.

After removing Q1_3, following *a priori* psychometric criteria plus content review, the model was re-estimated with the same four-factor structure. The exclusion of Q1_3 resolved the inadmissible negative residual variance associated with this item and yielded an admissible CFA solution while retaining acceptable global fit indices (Table 2, modified model). Standardized factor loadings remained strong and coherent within each factor: Attitudes: *λ* = 0.38–0.84; Norms: *λ* = 0.54–0.93; Confidence: *λ* = 0.81–0.86; Intentions: *λ* = 0.23–0.96. Item Q2_16, although showing a lower loading (*λ* = 0.23), displayed no floor or ceiling effects, contributed significantly to the factor (*p* < 0.001), and did not introduce estimation problems. Given its theoretical relevance to situational intentions, it was retained.

Latent factor correlations were moderate to high (r = 0.37–0.78, all *p* < 0.001), indicating that the subscales are meaningfully related while remaining empirically distinct, thereby supporting the structural coherence of the French I2A. Although the chi-square test was statistically significant, this result was expected given the large number of questionnaire items and the estimation method used for ordinal Likert-type responses.

### Scale descriptive statistics

Table 3 presents descriptive statistics and internal consistency estimates for the final I2A-French subscales. Internal consistency was good for Intentions (*α* = 0.86; *ω* = 0.87), Norms (*α* = 0.88; *ω* = 0.90), and Confidence (*α* = 0.87; *ω* = 0.89). The Attitudes subscale showed moderate Cronbach’s alpha (*α* = 0.68); however, McDonald’s omega indicated satisfactory reliability (*ω* = 0.75), despite unequal item contributions within the subscale. Because items within the Attitudes subscale contributed unevenly to the construct, omega was considered a more appropriate reliability indicator.

**Table 3:** Scale descriptive statistics.

**Subscale**	**Items**	**Scoring** **range**	**Min**	**Max**	**Mean**	**SD**	**Cronbach's *α***	**McDonald's *ω***
Intentions	11	11–55	11	55	49	6.03	0.86	0.87
Attitudes	8	8–40	12	40	35	3.72	0.66	0.75
Norms	7	7–35	7	35	31	4.48	0.87	0.90
Confidence	7	7–35	7	35	31.6	4.32	0.87	0.89

*Note*. Subscale scores were computed by summing item responses within each factor. Missing data were minimal (<1.4% per item) and were imputed using item means for subscale scoring only. Cronbach’s alpha was computed on imputed data.

### Theoretical coherence within the Theory of Planned Behavior framework

To further examine whether associations between constructs were consistent with the Theory of Planned Behavior framework, linear regression analyses were conducted to explore associations between CPR training exposure and I2A subscales. The number of prior CPR trainings was self-reported and treated as a continuous predictor variable. Each additional prior CPR training reported was associated with an average 0.49-point increase in Confidence score (*β* = 0.49, *p* = 0.002), although the proportion of explained variance remained modest (2.6%). In contrast, the number of training courses was not significantly associated with Intentions (*β* = 0.13, *p* = 0.55).

A multiple regression model was then used to examine whether relationships between Attitudes, Norms, Confidence, and Intentions were consistent with the Theory of Planned Behavior framework. Together, these variables explained 59% of the variance in Intentions (adjusted *R*^2^ = 0.59, *p* < 0.001). Confidence showed the strongest association with Intentions (*β* = 0.76, *p* < 0.001), followed by Norms (*β* = 0.42, *p* < 0.001). Attitudes were not significantly associated with Intentions after accounting for the other constructs (*β* = −0.04, *p* = 0.47). Collinearity between variables remained low, indicating that each construct contributed independently to the model.

## Discussion

The original four-factor structure of the I2A was successfully replicated in the French version using parcel-based CFA, and the item-level analysis further confirmed this structure. The removal of item Q1_3, due to extreme ceiling effects and estimation problems, improved model stability without altering the theoretical structure of the instrument. Reliability estimates supported adequate internal consistency across subscales. McDonald’s omega was particularly informative for the Attitudes factor because items contributed unevenly to this construct. Overall, the French I2A demonstrates sound psychometric properties and structural robustness. Additional research is indicated to measure the I2A-French with and without Q1_3 to determine if this is an artifact of the population surveyed.

We did not compare subgroups (e.g., first responders volunteers vs students) because the primary aim was scale-level validation; such comparisons will be important in future work. Consistent with the developmental strategy of the original I2A study, the present validation was intentionally conducted in a CPR-trained population before future evaluation in broader lay populations. Descriptive statistics showed high mean scores across subscales, approaching the upper limits of their ranges. This likely reflects the highly trained nature of the sample, as over half of participants reported five or more CPR courses. Similar ceiling patterns have been reported in other CPR-trained populations.^18^ In such contexts, willingness and related affective constructs may already be near ceiling. In highly trained samples, ceiling effects may reduce variability in item responses, attenuate the precision of CFA parameter estimation, inflate internal consistency estimates, and limit sensitivity to individual differences or pre-post changes. Future research should therefore consider the development or adaptation of a more lay-oriented version of the I2A capable of capturing greater variability in populations with limited prior CPR exposure.

Beyond structural validity, the observed associations between constructs support the theoretical coherence of the instrument within a Theory of Planned Behavior framework. Prior CPR training was associated with higher Confidence but not directly with Intentions, consistent with models suggesting that mastery experiences strengthen perceived self-efficacy,^19^ which in turn influences intention to act.^20^ When examined simultaneously, Confidence emerged as the strongest predictor of Intentions, with Norms contributing independently, whereas Attitudes showed no unique association.

In trained populations, self-efficacy and normative influences may therefore be more discriminating determinants of intention than attitudinal evaluations, particularly when attitudes toward CPR are already uniformly positive. Importantly, this pattern is consistent with the Reasoned Action Approach, which posits that the relative contribution of attitudes, norms, and perceived control may vary depending on the behavior and the population under study. Fishbein^21^ explicitly notes that some behaviors are primarily attitudinally driven, whereas others are more strongly shaped by normative or control considerations, and that this balance may differ across cultural contexts. In the present Swiss CPR-trained sample, intention appears to be predominantly under self-efficacy and normative control rather than attitudinal influence. This finding reinforces the importance of empirically identifying the dominant determinants of intention within each target population rather than assuming invariant Theory of Planned Behavior pathways across contexts. Because all Theory of Planned Behavior constructs were measured within the same instrument and dataset, these findings primarily support internal theoretical coherence rather than external predictive validity or association with actual bystander behavior.

The predominance of Confidence and Norms suggests that resuscitation education programs in trained populations should prioritize experiential methods that strengthen self-efficacy and address normative expectations among peers and organizations. Such approaches may help translate CPR training into actual bystander intervention during out-of-hospital cardiac arrest. These findings highlight the need for affective-domain tools like I2A to evaluate training in underserved groups, aligning with ILCOR calls to address disparities.^2^

This Swiss context also introduces limitations. High baseline training exposure and relative cultural homogeneity may limit generalizability beyond Swiss French-speaking CPR-trained populations, particularly to less-trained or more socio-culturally diverse groups. Longitudinal and intervention studies are needed to examine sensitivity to change and to test the proposed “*Training → Confidence → Intentions*” pathway. This study represents an initial step toward monitoring the effectiveness of ERC-aligned community training initiatives by identifying affective barriers to lay responder CPR and AED use in French-speaking regions. Such affective-domain monitoring aligns with ERC recommendations to evaluate not only skills and knowledge but also behavioral determinants that influence real-world bystander intervention. The availability of a validated French-language instrument may facilitate future studies examining how educational strategies influence bystander CPR response within regional OHCA systems.

## Conclusion

This study provides a first psychometric validation of the I2A in French-speaking CPR-trained populations, confirming its four-factor structure, internal consistency, and theoretical coherence within a Theory of Planned Behavior framework. The instrument appears suitable for evaluating affective-domain outcomes in trained responder groups and for distinguishing the relative contribution of confidence, norms, and attitudes to intention.

Future studies should evaluate predictive validity, responsiveness to training, and applicability in more heterogeneous and less-trained populations.

## CRediT authorship contribution statement

**Marco Pedrotti:** Writing – review & editing, Writing – original draft, Visualization, Supervision, Project administration, Methodology, Investigation, Funding acquisition, Formal analysis, Data curation, Conceptualization. **Baptiste Lucien:** Writing – review & editing, Project administration, Methodology, Investigation, Funding acquisition, Formal analysis, Data curation, Conceptualization. **Philippe Terrier:** Writing – review & editing, Methodology, Funding acquisition, Formal analysis, Data curation. **Jérémy Torrent:** Writing – review & editing, Investigation, Data curation. **Sandrine Dénéréaz:** Writing – review & editing, Project administration, Investigation. **Thierry Spichiger:** Writing – review & editing, Project administration, Investigation. **Christophe Bélet:** Writing – review & editing, Project administration, Investigation. **Jeffrey L. Pellegrino:** Writing – review & editing, Writing – original draft, Supervision, Methodology, Funding acquisition, Conceptualization.

## Funding

This work was supported by the University of Applied Sciences and Arts Western Switzerland HES-SO (grant no. 136979).

## Data availability

The data supporting the findings of this study are available in the supplementary materials.

## Declaration of generative AI and AI-assisted technologies in the manuscript preparation process

During the preparation of this work the authors used ChatGPT in order to translate content from French to English and to rephrase text they had originally written in English by themselves. After using this tool, the authors reviewed and edited the content as needed and take full responsibility for the content of the publication.

## Declaration of competing interest

The authors declare that they have no known competing financial interests or personal relationships that could have appeared to influence the work reported in this paper.

## References

[1] Greif R., Lauridsen K.G., Djärv T., Ek J.E., Monnelly V., Monsieurs K.G. (2025). European Resuscitation Council guidelines 2025 executive summary. Resuscitation.

[2] Ko Y.C., Hsieh M.J., Schnaubelt S., Matsuyama T., Cheng A., Greif R. (2023). Disparities in layperson resuscitation education: a scoping review. Am J Emerg Med.

[3] Minna S., Leena H., Tommi K. (2022). How to evaluate first aid skills after training: a systematic review. Scand J Trauma Resusc Emerg Med.

[4] Cheng A., Nadkarni V.M., Mancini M.B., Hunt E.A., Sinz E.H., Merchant R.M. (2018). Resuscitation education science: educational strategies to improve outcomes from cardiac arrest: a scientific statement from the American Heart Association. Circulation.

[5] Lim X.M.A., Liao W.A., Wang W., Seah B. (2022). The effectiveness of technology-based cardiopulmonary resuscitation training on the skills and knowledge of adolescents: systematic review and meta-analysis. J Med Internet Res.

[6] Miller B., Pellegrino J.L. (2018). Measuring intent to aid of lay responders: survey development and validation. Health Educ Behav.

[7] Interverband für Rettungswesen, Swiss Resuscitation Council. SWISSRECA Rapport annuel 2024; 2025. Available from https://www.144.ch/fr/swissreca-jahresbericht-2024/.

[8] Mokkink LB, Prinsen CAC, Patrick DL, Alonso J, Bouter LM, de Vet HCW, et al. COSMIN study design checklist for patient-reported outcome measurement instruments; 2019. Available from https://www.cosmin.nl/finding-right-tool/conducting-study-measurement-properties/.

[9] von Elm E., Altman D.G., Egger M., Pocock S.J., Gøtzsche P.C., Vandenbroucke J.P. (2007). Strengthening the Reporting of Observational Studies in Epidemiology (STROBE) statement: guidelines for reporting observational studies. BMJ.

[10] Sousa V.D., Rojjanasrirat W. (2011). Translation, adaptation and validation of instruments or scales for use in cross-cultural health care research: a clear and user-friendly guideline. J Eval Clin Pract.

[11] Harris P.A., Taylor R., Minor B.L., Elliott V., Fernandez M., O’Neal L. (2019). The REDCap consortium: building an international community of software platform partners. J Biomed Inform.

[12] Harris P.A., Taylor R., Thielke R., Payne J., Gonzalez N., Conde J.G. (2009). Research electronic data capture (REDCap)—a metadata-driven methodology and workflow process for providing translational research informatics support. J Biomed Inform.

[13] Brown T.A. (2015).

[14] Cheung G.W., Rensvold R.B. (2002). Evaluating goodness-of-fit indexes for testing measurement invariance. Struct Equ Model.

[15] Sharma S., Mukherjee S., Kumar A., Dillon W.R. (2005). A simulation study to investigate the use of cutoff values for assessing model fit in covariance structure models. J Bus Res.

[16] Hooper D., Coughlan J., Mullen M.R. (2008). Structural equation modelling: guidelines for determining model fit. Electron J Bus Res Methods.

[17] Kline R.B. (2016).

[18] Mohd Hashim N.I., Daud A., Mohammed Nawi A. (2025). Factors influencing willingness to perform cardiopulmonary resuscitation (CPR) and use an automated external defibrillator (AED) among non-healthcare community participants in a CPR fun run. BMC Public Health.

[19] Bandura A. (1977). Self-efficacy: toward a unifying theory of behavioral change. Psychol Rev.

[20] Panchal A.R., Fishman J., Camp-Rogers T., Starodub R., Merchant R.M. (2015). An “intention-focused” paradigm for improving bystander CPR performance. Resuscitation.

[21] Fishbein M. (2008). A reasoned action approach to health promotion. Med Decis Mak Int J Soc Med Decis Mak.

