# Supplementary material for: Translation and psychometric validation of the Intent-to-Aid survey for bystander response among French-speaking CPR-trained populations
Source: Resusc Plus. 2026 Jun 29;30:101400. doi: 10.1016/j.resplu.2026.101400 (PMC13377492; doi:10.1016/j.resplu.2026.101400)
Supplement: Supplementary Data 5 [file mmc5.pdf]

Bonjour,

Merci de participer à l'étude sur l'intention d'aider en cas de réanimation cardio-pulmonaire (RCP).

Vos réponses, anonymes et confidentielles, seront utilisées uniquement à des fins de recherche.

La participation est volontaire et anonyme et vous pouvez arrêter à tout moment, sans conséquence.

En poursuivant et en validant ce questionnaire, vous indiquez avoir compris les informations ci-dessus et acceptez d'y participer.

Vous allez maintenant lire une série d'affirmations. Pour chacune d'elles, indiquez dans quelle mesure vous êtes d'accord ou non, en sélectionnant un chiffre entre 1 (totalement en désaccord) et 5 (totalement d'accord).Il n'y a pas de bonne ou de mauvaise réponse - ce qui nous importe, c'est votre perception personnelle.

On parle d'une urgence nécessitant des premiers secours lorsqu'une action immédiate, y compris la RCP, est nécessaire pour sauver une vie, réduire ou stopper la souffrance, prévenir une aggravation de la maladie ou de la blessure, et favoriser le rétablissement.

Pour chaque affirmation, veuillez indiquer votre niveau d'accord :

	Totalement en désaccord	Plutôt en désaccord	Ni d'accord ni en désaccord	Plutôt d'accord	Totalement d'accord
En général, les gens s'attendent à ce que j'aide en cas d'urgence nécessitant des premiers secours.	○	○	○	○	○

	Totalement en désaccord	Plutôt en désaccord	Ni d'accord ni en désaccord	Plutôt d'accord	Totalement d'accord
Tout le monde a la responsabilité d'aider en cas d'urgence nécessitant des premiers secours.	○	○	○	○	○

Je veux bien me porter volontaire pour aider ______________ en cas d'urgence nécessitant des premiers secours.

	Totalement en désaccord	Plutôt en désaccord	Ni d'accord ni en désaccord	Plutôt d'accord	Totalement d'accord
un membre de ma famille	○	○	○	○	○
une connaissance	○	○	○	○	○
une personne inconnue	○	○	○	○	○
une personne qui semble sale (p. ex. vêtements souillés, odeur désagréable)	○	○	○	○	○

une personne ensanglantée	○	○	○	○	○

Je ferais usage de mes compétences de réanimation cardio-pulmonaire (RCP)/premiers secours en cas d'urgence

	Totalement en désaccord	Plutôt en désaccord	Ni d'accord ni en désaccord	Plutôt d'accord	Totalement d'accord
s'il y avait d'autres personnes sur le lieu de l'urgence (qui ne sont pas certifiées en RCP).	○	○	○	○	○
si quelqu'un s'étouffait.	○	○	○	○	○
si je devais faire du bouche-à-bouche à la victime.	○	○	○	○	○
si j'avais un masque ou une barrière respiratoire (p.ex. masque de poche/pocket mask).	○	○	○	○	○
si une personne s'écroulait devant moi.	○	○	○	○	○
si je trouvais une personne paraissant bleue ou froide.	○	○	○	○	○
même si je pensais que je risquais d'avoir des ennuis ou d'être en poursuite judiciaire.	○	○	○	○	○

Je me sens en confiance sur le fait que je pourrais

	Totalement en désaccord	Plutôt en désaccord	Ni d'accord ni en désaccord	Plutôt d'accord	Totalement d'accord
appeler efficacement le 144 ou de trouver de l'aide supplémentaire en cas d'urgence.	○	○	○	○	○
effectuer efficacement des tapes dorsales (manœuvre de désobstruction).	○	○	○	○	○
effectuer efficacement des compressions abdominales (manœuvre de Heimlich).	○	○	○	○	○
déterminer efficacement si une personne nécessite une RCP.	○	○	○	○	○
effectuer efficacement une RCP si j'avais une brève liste d'instructions.	○	○	○	○	○
effectuer efficacement une RCP si quelqu'un m'apportait de l'aide.	○	○	○	○	○
effectuer une RCP efficacement en autonomie.	○	○	○	○	○

Je pars du principe que

	Totalement en désaccord	Plutôt en désaccord	Ni d'accord ni en désaccord	Plutôt d'accord	Totalement d'accord
une victime d'arrêt cardiaque souhaiterait que je pratique une RCP sur elle.	○	○	○	○	○
la famille d'une victime d'arrêt cardiaque souhaiterait que je pratique une RCP sur la victime.	○	○	○	○	○
ma famille souhaiterait que je pratique une RCP sur une victime d'arrêt cardiaque.	○	○	○	○	○
d'autres témoins / personnes à proximité souhaiteraient que je pratique une RCP sur une victime d'arrêt cardiaque.	○	○	○	○	○
d'autres personnes ne se trouvant pas à proximité souhaiteraient que je pratique une RCP sur une victime d'arrêt cardiaque.	○	○	○	○	○

	Totalement d'accord	Plutôt d'accord	Ni d'accord ni en désaccord	Plutôt en désaccord	Totalement en désaccord
En considérant votre formation, la RCP est difficile à apprendre.	○	○	○	○	○

Dans cette section, vous allez lire la description d'une situation d'urgence. Imaginez comment vous pourriez vous sentir et agir dans cette situation, puis répondez aux questions ci-dessous. Il n'y a pas de bonnes ou de mauvaises réponses :

"Vous et un·e ami·e êtes à un concert dans un grand stade. Le stade est presque plein. En milieu de concert, vous allez chercher une boisson. Alors que vous faites la file, votre ami·e s'effondre et semble inconscient·e."

	Pas du tout probable	Peu probable	Moyennement probable	Assez probable	Très probable
Quelle est la probabilité que vous demandiez à quelqu'un d'appeler de l'aide, ou plus spécifiquement le 144 ?	○	○	○	○	○

	Pas du tout probable	Peu probable	Moyennement probable	Assez probable	Très probable
Quelle est la probabilité que vous vérifiez les mouvements ou la respiration d'une personne avec qui vous partagez un lien d'amitié ?	○	○	○	○	○

	Pas du tout nerveux(se)	Un peu nerveux(se)	Modérément nerveux(se)	Assez nerveux(se)	Très nerveux(se)
A quel point seriez-vous en état de nervosité à l'idée d'utiliser un défibrillateur automatisé externe (DAE) dans cette situation ?	◯	◯	◯	◯	◯

	Pas du tout probable	Peu probable	Moyennement probable	Assez probable	Très probable
Quelle est la probabilité que vous utilisiez un DAE sur une personne avec qui vous partagez un lien d'amitié ?	◯	◯	◯	◯	◯

	Pas du tout confiant(e)	Peu confiant(e)	Assez confiant(e)	Confiant(e)	Très confiant(e)
À quel point vous sentez-vous en confiance sur le fait de pouvoir placer les électrodes du DAE aux bons endroits ?	◯	◯	◯	◯	◯

	Pas du tout probable	Peu probable	Moyennement probable	Assez probable	Très probable
Quelle est la probabilité que vous appuyiez sur le bouton < < choc >> ?	◯	◯	◯	◯	◯

	Pas du tout confiant(e)	Peu confiant(e)	Assez confiant(e)	Confiant(e)	Très confiant(e)
À quel point vous sentez-vous en confiance sur le fait de pouvoir appuyer sur le bouton de choc au bon moment ?	◯	◯	◯	◯	◯

Merci pour vos réponses ! Le questionnaire est bientôt fini : il reste seulement quelques questions d'ordre général. Pour chacune, vous pouvez toujours sélectionner "Je préfère ne pas répondre" si une question ne vous convient pas.

Combien de cours de réanimation cardio-pulmonaire (RCP) / défibrillateur automatisé externe (DAE) avez-vous suivis au total ?

- ◯ Aucun
- ◯ 1
- ◯ 2
- ◯ 3
- ◯ 4
- ◯ 5 ou plus
- ◯ Je préfère ne pas répondre

Quel est le plus haut niveau d'études que vous avez atteint ?

- ◯ Aucune formation terminée
- ◯ Formation secondaire II (CFC, École de culture Générale, ou équivalent)
- ◯ Formation professionnelle supérieure (ES, brevet ou diplôme fédéral)
- ◯ Bachelor (Université / HES / HEP)
- ◯ Master (Université / HES / HEP)
- ◯ Doctorat
- ◯ Je préfère ne pas répondre

Quelle est votre origine ? (plusieurs réponses possibles)	☐ Africaine<br>☐ Amérindienne<br>☐ Asiatique<br>☐ Caucasienne / Européenne<br>☐ Je préfère ne pas répondre<br>☐ Autre
Si autre, veuillez préciser:	__________________________________
Quelle est votre langue maternelle ? (plusieurs réponses possibles)	☐ Albanais<br>☐ Allemand<br>☐ Anglais<br>☐ Croate<br>☐ Espagnol<br>☐ Français<br>☐ Italien<br>☐ Portugais<br>☐ Romanche<br>☐ Serbe<br>☐ Autre<br>☐ Je préfère ne pas répondre
Si autre, veuillez préciser:	__________________________________
Quel est votre sexe ?	☐ Femme<br>☐ Homme<br>☐ Autre / je ne me reconnais pas dans ces catégories<br>☐ Je préfère ne pas répondre
Quel est votre age ?<br><br>(vous pouvez laisser vide si vous préférez ne pas répondre)	__________________________________
Pour remercier les participants, un cours BLS-AED-SRC gratuit sera offert à 8 personnes.<br>Le certificat délivré est valable 2 ans selon les directives du Swiss Resuscitation Council.<br><br>Si vous souhaitez participer au tirage au sort, veuillez indiquer votre adresse e-mail ; sinon, laissez simplement le champ vide.<br><br>Pour toute question vous pouvez nous contacter aux adresses e-mail suivantes :<br>baptiste.lucien@he-arc.ch ou marco.pedrotti@he-arc.ch	__________________________________
