# Supplementary material for: Translation and psychometric validation of the Intent-to-Aid survey for bystander response among French-speaking CPR-trained populations
Source: Resusc Plus. 2026 Jun 29;30:101400. doi: 10.1016/j.resplu.2026.101400 (PMC13377492; doi:10.1016/j.resplu.2026.101400)
Supplement: Supplementary Data 6 [file mmc6.docx]

| **Q5_1** |
| --- |
| En général, les gens s’attendent à ce que j’aide en cas d’urgence nécessitant des premiers secours. |
| **Q5_4** |
| Tout le monde a la responsabilité d’aider en cas d’urgence nécessitant des premiers secours. |
| **Q1_1** |
| Je veux bien me porter volontaire pour aider un membre de ma famille en cas d’urgence nécessitant des premiers secours. |
| **Q1_4** |
| Je veux bien me porter volontaire pour aider une personne inconnue en cas d’urgence nécessitant des premiers secours. |
| **Q1_5** |
| Je veux bien me porter volontaire pour aider une personne qui semble sale (p. ex. vêtements souillés, odeur désagréable) lors d’une urgence nécessitant des premiers secours. |
| **Q1_6** |
| Je veux bien me porter volontaire pour aider une personne ensanglantée en cas d’urgence nécessitant des premiers secours. |
| **Q2_1** |
| Je ferais usage de mes compétences de réanimation cardio-pulmonaire (RCP)/premiers secours en cas d’urgence s’il y avait d’autres personnes sur le lieu de l’urgence (qui ne sont pas certifiées en RCP). |
| **Q2_7** |
| Je ferais usage de mes compétences en RCP/premiers secours si quelqu’un s’étouffait. |
| **Q2_16** |
| Je ferais usage de mes compétences en RCP/premiers secours en cas d’urgence si je devais faire du bouche-à-bouche à la victime. |
| **Q2_6** |
| Je ferais usage de mes compétences en RCP/premiers secours si j’avais un masque ou une barrière respiratoire (p.ex. masque de poche/pocket mask). |
| **Q2_3** |
| Je ferais usage de mes compétences en RCP/premiers secours en cas d’urgence si une personne s’écroulait devant moi. |
| **Q2_13** |
| Je ferais usage de mes compétences en RCP/premiers secours si je trouvais une personne paraissant bleue ou froide. |
| **Q2_4** |
| Je ferais usage de mes compétences en RCP/premiers secours en cas d’urgence même si je pensais que je risquais d’avoir des ennuis ou d’être en poursuite judiciaire. |
| **Q4_1** |
| Je me sens en confiance sur le fait que je pourrais appeler efficacement le 144 ou de trouver de l’aide supplémentaire en cas d’urgence. |
| **Q4_6** |
| Je me sens en confiance sur le fait que je pourrais effectuer efficacement des tapes dorsales (manœuvre de désobstruction). |
| **Q4_7** |
| Je me sens en confiance sur le fait que je pourrais effectuer efficacement des compressions abdominales (manœuvre de Heimlich). |
| **Q4_2** |
| Je me sens en confiance sur le fait que je pourrais déterminer efficacement si une personne nécessite une RCP. |
| **Q4_3** |
| Je me sens en confiance sur le fait que je pourrais effectuer efficacement une RCP si j’avais une brève liste d’instructions. |
| **Q4_5** |
| Je me sens en confiance sur le fait que je pourrais effectuer efficacement une RCP si quelqu’un m’apportait de l’aide. |
| **Q4_8** |
| Je me sens en confiance sur le fait que je pourrais effectuer une RCP efficacement en autonomie. |
| **Q6_1** |
| Je pars du principe qu’une victime d'arrêt cardiaque souhaiterait que je pratique une RCP sur elle. |
| **Q6_2** |
| Je pars du principe que la famille d’une victime d'arrêt cardiaque souhaiterait que je pratique une RCP sur la victime. |
| **Q6_4** |
| Je pars du principe que ma famille souhaiterait que je pratique une RCP sur une victime d'arrêt cardiaque. |
| **Q6_3** |
| Je pars du principe que d’autres témoins / personnes à proximité souhaiteraient que je pratique une RCP sur une victime d'arrêt cardiaque. |
| **Q6_6** |
| Je pars du principe que d’autres personnes ne se trouvant pas à proximité souhaiteraient que je pratique une RCP sur une victime d'arrêt cardiaque. |
| **Q59_5** |
| En considérant votre formation, la RCP est difficile à apprendre. |
|  |
| Dans cette section, vous allez lire la description d'une situation d'urgence. Imaginez comment vous pourriez vous sentir et agir dans cette situation, puis répondez aux questions ci-dessous. Il n'y a pas de bonnes ou de mauvaises réponses :  "Vous et un·e ami·e êtes à un concert dans un grand stade. Le stade est presque plein. En milieu de concert, vous allez chercher une boisson. Alors que vous faites la file, votre ami·e s'effondre et semble inconscient·e." |
| **Q35** |
| Quelle est la probabilité que vous demandiez à quelqu’un d’appeler de l’aide, ou plus spécifiquement le 144 ? |
| **Q36** |
| Quelle est la probabilité que vous vérifiez les mouvements ou la respiration d’une personne avec qui vous partagez un lien d’amitié ? |
| **Q37** |
| A quel point seriez-vous en état de nervosité à l’idée d’utiliser un défibrillateur automatisé externe (DAE) dans cette situation ? |
| **Q45** |
| Quelle est la probabilité que vous utilisiez un DAE sur une personne avec qui vous partagez un lien d’amitié ? |
| **Q40** |
| À quel point vous sentez-vous en confiance sur le fait de pouvoir placer les électrodes du DAE aux bons endroits ? |
| **Q41** |
| Quelle est la probabilité que vous appuyiez sur le bouton « choc » ? |
| **Q42** |
| À quel point vous sentez-vous en confiance sur le fait de pouvoir appuyer sur le bouton de choc au bon moment ? |

The following item has been removed from the French version:

| **Q1_3** |
| --- |
| Je veux bien me porter volontaire pour aider une connaissance en cas d’urgence nécessitant des premiers secours. |
